# Tuberculosis infection screening in children with close contact: a hospital-based study

**DOI:** 10.1186/s12879-021-06480-2

**Published:** 2021-08-13

**Authors:** Lin Sun, Xue Qi, Yajie Guo, Hui Qi, Jieqiong Li, Xirong Wu, Qingqin Yin, Yan Guo, Baoping Xu, Yacui Wang, Qi Jin, Lei Gao, Adong Shen

**Affiliations:** 1 National Clinical Research Center for Respiratory Diseases, National Key Discipline of Pediatrics, Capital Medical University, Key Laboratory of Major Diseases in Children, Ministry of Education, Beijing Children’s Hospital, Capital Medical University, National Center for Children’s Health, Beijing, China; 2 MOH Key Laboratory of Systems Biology of Pathogens, Institute of Pathogen Biology, and Centre for Tuberculosis, Chinese Academy of Medical Sciences and Peking Union Medical College, Beijing, China; 3 Department of Respiratory, Qilu Children’s Hospital of Shandong University, Jinan, China

**Keywords:** Latent tuberculosis infection, Children, Contact risk

## Abstract

**Background:**

Identifying and prioritizing at-risk populations is critical for pediatric tuberculosis control. We aimed to identify a latent tuberculosis infection (LTBI) screening strategy that is appropriate for the Chinese context among children with different TB exposure levels and to explore its clinical importance.

**Methods:**

During 2013–2015, we enrolled hospitalized children with suspected respiratory infectious disease (RID) for LTBI screening using the tuberculin skin test (TST) and interferon-γ release assay (IGRA) T-SPOT.TB as part of a work up for their RID. Participants with confirmed diagnosis were classified into three subgroups according to level of exposure to TB: no reported contact risk, with household contact risk, and with non-household contact risk.

**Results:**

A total 6202 children (median age: 4.76 years; interquartile range: 1.0–8.0 years) were enrolled. Children with no reported contact risk had the lowest proportions of positive results for the IGRA (0.7%) and TST (3.3%). The proportion of positive results for each test was higher for household contacts than non-household contacts. The TST positive proportion was much higher than that for the IGRA in all three groups. Children with IGRA+/TST+ results had larger indurations than those with IGRA− /TST+  results (15 mm vs. 13 mm, *P* = 0.02). For IGRA, older age (> 5 years) and non-household or household contact risk were associated with a positive result.

**Conclusions:**

Positive IGRA results in children with a contact risk can serve as a critical reference for LTBI management. IGRA can be used, in preference to TST, for Chinese children with a TB exposure risk.

**Supplementary Information:**

The online version contains supplementary material available at 10.1186/s12879-021-06480-2.

## Background

Tuberculosis (TB) is a major threat to children’s health [1]. In 2019, approximately 10 million people fell ill with TB globally and 1.2 million people died. Children carry nearly 12% of the global TB disease burden. Owing to their immature immune systems, children are more likely to develop active disease after infection with *Mycobacterium tuberculosis* (MTB) [2, 3]; the 5-year risk is 33% in children < 5 years old [4]. In addition, the risk of children being infected with MTB is associated with the degree of exposure. Children with household contact have a higher infection risk than those with a history of community exposure [5]. Therefore, improving latent tuberculosis infection (LTBI) and active TB management in children with MTB exposure is critical for TB control.

The World Health Organization (WHO) provides guidelines for identifying and prioritizing at-risk populations for targeted interventions of LTBI testing and treatment [6, 7]. Updated guidelines on the management of LTBI recommend that either a tuberculin skin test (TST) or interferon-gamma release assay (IGRA) can be used to test for LTBI [7]. Considering the cost of LTBI testing and treatment, the influence of Bacillus Calmette–Guérin (BCG) vaccination and necessary laboratory conditions for these testing methods, the LTBI screening strategy varies among different counties. For example, a TST-only strategy has been used in France whereas the combined use of IGRA and TST has been applied in Germany, comprising an IGRA following a positive TST result, for contact investigation among children [8].

China is among the countries with the largest global TB burden, with an incidence rate per capita of 61 per 100,000 [1]. The prevalence of MTB infection varies nationwide but it is much higher in western than in eastern regions of China, such as in Beijing. Data from the fourth population-based nationwide epidemiological investigation in 2000 showed that 9% of children were MTB infected, using a TST-only screening strategy [9]. Therefore, early identification of children with LTBI and timely administration of prophylactic treatment are important for TB control in children.

In recent years, the Chinese government has established several regulations for TB prevention and control, which recommend surveillance for active TB and LTBI in at-risk populations including those with a TB contact history, such as students who have had contact with active TB in school or children with immunosuppressive diseases. In 2020, in order to promote the work of TB control in children, a national guideline was released requiring screening of active TB and LTBI using the TST in all students during their first year of admission to kindergarten, primary and secondary schools, and university irrespective of their contact history. The scheme includes collecting information to identify a history of close contact with active TB as well as symptoms, and conducting TST in students with TB contact history, and then performing chest X-ray in those with a positive TST result or with TB symptoms. The LTBI burden among children in China has not been systematically evaluated in recent years. More importantly, the efficacy of the tests used to screen LTBI and choice of the IGRA or TST in at-risk children have not been clearly evaluated.

Discordant results between the IGRA and TST are often observed in LTBI screening of children. How to interpret the clinical importance of discordant results and how to manage children with IGRA− /TST+  or IGRA+ /TST− results remain unclear. Therefore, we conducted an evaluation study to analyze the performance of the IGRA and TST in a pediatric population with varying levels of MTB exposure. IGRA and TST was being performed as a component of a preliminary TB disease evaluation in children admitted with respiratory symptoms. In this study, the IGRA and TST were evaluated individually and in combination. Investigation of the combined use of these two tests is critical, to support the adequate management of LTBI among children in countries like China.

## Methods

### Study design and participants

Children with suspected RID were enrolled in the study to screen LTBI at a tertiary care hospital Beijing Children’s Hospital between January 1, 2013 and December 31, 2015. Children were included furtherly if they were (i) diagnosed with RID; (ii) younger than 18 years old; and (iii) tested using an IGRA T-SPOT.TB (TSpot; Oxford Immunotec, Oxford, UK) and TST. Children with respiratory infectious disease (RID) were defined as those excluded active TB but with respiratory symptoms and confirmed etiological evidence of infection with virus, mycoplasma, or bacteria. Patients not tested using these two methods were excluded from further analysis. Because impaired immunity may induce false negative results and produce biases, children who were HIV positive were also excluded. Because of the high TB burden in China, the exact source of MTB infection is difficult to ascertain. So if parents give informed consents, all the children with RID will be suggested to be tested by IGRA or TST to assistant the diagnosis of active TB. Active TB was further screened in children with (i) positive TST or IGRA results; (ii) an exposure history to active TB; or (iii) radiographic evidence consistent with TB. Children were clinically diagnosed with active TB in accordance with the standards previously reported [10].

This research was approved by the Ethics Committee of Beijing Children’s Hospital (2018–96). Written informed consent was obtained from their guardians.

### Procedures

All methods were performed in accordance with the relevant guidelines and regulations. Venous blood was collected for TSpot and then processed according to the manufacturer’s recommendations. The children with borderline TSpot results were retested within one week. If a positive or a negative result was got in the second test, the result of the retesting was recorded and analyzed in the study. If the retest still presented as borderline result, the result was recorded as borderline result. The TST was performed using an intradermal injection of 5-IU purified protein derivative (PPD) from the Chengdu Institute of Biological Products (Chengdu, China). Trained pediatricians measured the transverse induration in millimeters 48–72 h later. A cutoff value ≥ 10 mm was used to define a positive TST result. Results using six well spots as the cutoff for the TSpot and ≥ 5 mm as the cutoff for the TST are shown in Additional file 1: Table S1.

Sociodemographic and clinical information was collected by two trained interviewers using structured electronic questionnaires. The data included age, sex, history of close contact with a person with active TB, BCG vaccination, and presence of a BCG scar. All children were classified into one of three subgroups based on their contact history: no reported contact risk, with a household contact risk, and with a non-household contact risk. Household contact risk was defined as household contact with a family member with TB and sharing an enclosed space with an index case for extended daytime periods (≥ 8 consecutive hours/day for several days) [11]. Non-household contact risk was defined as contact with TB cases outside of the household, such as at a social gathering or location outside the home.

### Statistical analyses

All data were analyzed with SPSS (version 13.0; SPSS Inc., Chicago, IL, USA). The Mann–Whitney U test with two-sided testing was used for continuous variables. Values were expressed as mean (standard deviation, SD) for normally distributed data and median and interquartile range (IQR) for non-normally distributed data. Categorical variable frequencies between participants by IGRA and TST status were compared with the Pearson’s χ^2^ test.

## Results

**Fig. 1:**
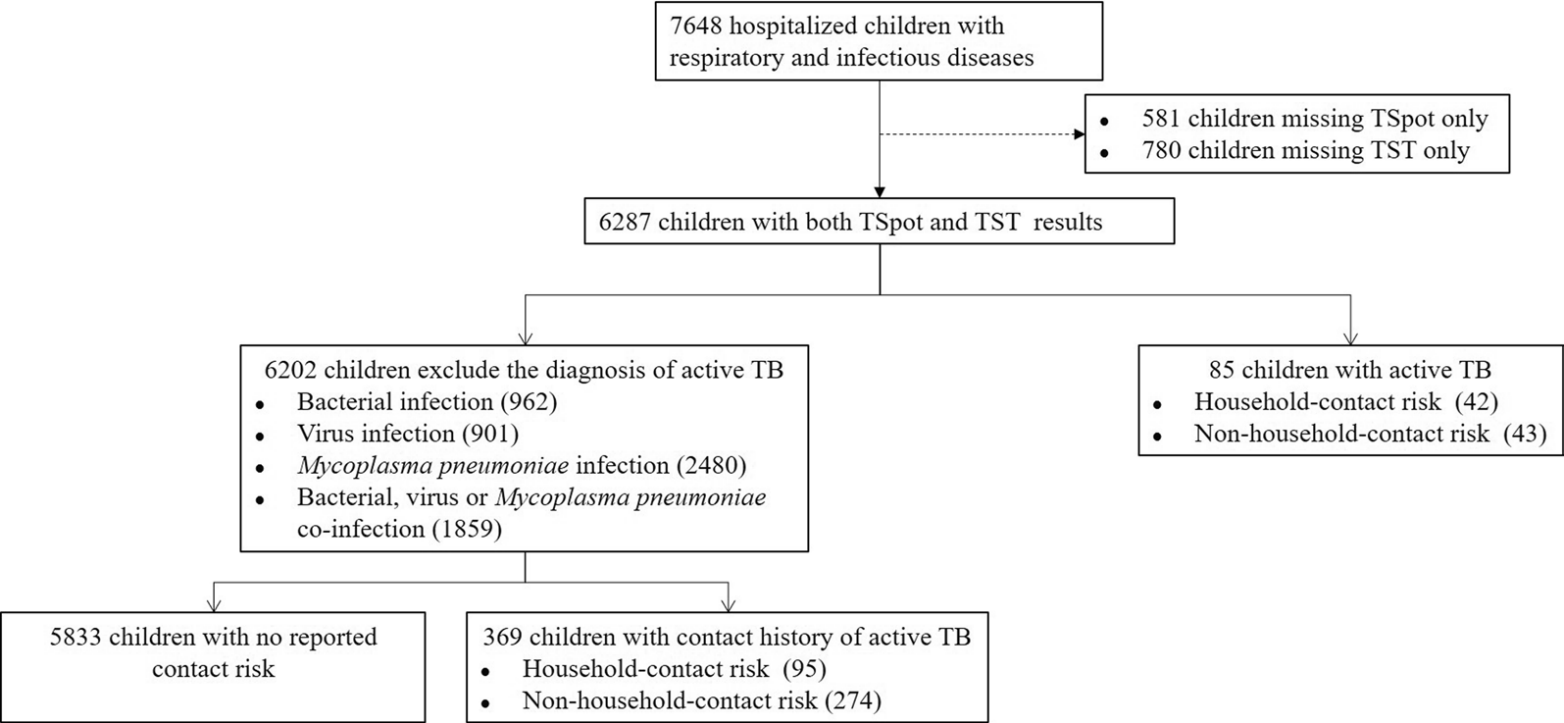
Flow chart of study population. TB, tuberculosis; Tspot, T-SPOT.TB; TST, tuberculin skin test

In this study, 7648 hospitalized children with suspected RID who were admitted to Beijing Children’s Hospital were screened for LTBI. Finally, we enrolled 6287 children with both IGRA and TST results available. Among them, 85 children were diagnosed with active TB (42 children had a contact history to active TB and 43 children had no contact history) and 6202 children were diagnosed with RID (369 children had a contact history to active TB and 5833 children had no contact history). Among 369 children with a self-reported contact history to active TB, 95 were classified in the household contact risk group and 274 in the non-household contact risk group (Fig. 1). The main characteristics of children with RID are shown in Table 1.

**Table 1:** Major characteristics of the study population as children with RID

	Total (n = 6202)	Subgroup with no reported contact risk (n = 5833)	Subgroup with non-household contact risk (n = 274)	Subgroup with household contact risk (n = 95)
Sex				
Male	3748 (60.4%)	3526 (60.4%)	161 (58.8%)	61 (64.2%)
Female	2454 (39.6%)	2307 (39.6%)	113 (41.2%)	34 (35.8%)
Age				
0–2y	2327 (37.5%)	2276 (39.0%)	40 (14.6%)	11 (11.6%)
3–4y	924 (14.9%)	872 (14.9%)	39 (14.2%)	13 (13.7%)
5–18	2951 (47.6%)	2685 (46.1%)	195 (71.2%)	71 (74.7%)
District				
Eastern	4662 (75.2%)	4443 (76.2%)	161 (58.8%)	58 (61.1%)
Middle	1042 (16.8%)	958 (16.4%)	66 (24.1%)	18 (18.9%)
Western	498 (8.0%)	432 (7.4%)	47 (17.2%)	19 (20.0%)
Resident place				
City	3376 (54.4%)	3185 (54.6%)	140 (51.1%)	51 (53.7%)
Rural	2826 (45.6%)	2648 (45.4%)	134 (48.9%)	44 (46.3%)
BCG vaccination history				
Yes	6086 (98.1%)	5722 (98.1%)	270 (98.5%)	94 (98.9%)
No	116 (1.9%)	111 (1.9%)	4 (1.5%)	1 (1.1%)
Presence of BCG scar				
Yes	5895 (95.0%)	5547 (95.1%)	260 (94.9%)	88 (92.6%)
No	307 (5.0%)	286 (4.9%)	14 (5.1%)	7 (7.4%)
IGRA positive results, n(%)	52 (0.8%)	41 (0.7%)	6 (2.2%)	5 (5.3%)
TST positive results, n(%)	191 (3.1%)	151 (3.3%)	23 (8.4%)	17 (17.9%)

Data are shown as n (%)

*RID* respiratory and infectious diseases, *BCG* Bacille Calmette Guerin, *IGRA* interferon-gamma release assay, *TST* tuberculin skin test

We determined the proportion of positive IGRA and TST results among 6202 children in whom active TB was ruled out according to different exposure levels. The lowest proportions of positive IGRA and TST results were observed in children with no reported contact risk (0.7% for IGRA and 3.3% for TST). The proportion of positive results for each test was higher for household contacts than non-household contacts, with 2.2% and 5.3% for IGRA in the non-household contact risk group and household contact risk group, respectively, and 8.4% and 17.9% for TST in the non-household contact and household contact risk group, respectively. Overall, the positive proportion for the TST was much higher than that for the IGRA in all three groups (Table 1).

Factors significantly associated with TST positivity were being a resident of the central and western regions of China and non-household or household contact with a patient who had active TB. For IGRA, older age (> 5 years), being a resident of the central and western regions of China and non-household or household contact risk were associated with a positive result (Table 2).

**Table 2:** Univariable and multivariable analysis of TST and IGRA positivity

	IGRA positivity (≥ 8 spots)			TST(≥ 10 mm)		
	n/N (%)	p for X^2^ test^#^	Adjusted OR*(95%CI)	n/N (%)	p for X^2^ test^#^	Adjusted OR*(95%CI)
Sex		0.32			0.98	
Male	35/3748 (0.9%)		Reference	115/3748 (3.1%)		Reference
Female	17/2454 (0.7%)		0.7 (0.4–1.2)	76/2454 (3.1%)		1.0 (0.8–1.3)
Age(years)		0.006			0.049	
0–2 y	12/2327 (0.5%)		Reference*	63/2327 (2.7%)		Reference*
3–4 y	4/924 (0.4%)		0.8 (0.3–2.2)*	21/924 (2.3%)		0.8 (0.5–1.2)
5–18	36/2951 (1.2%)		2.5 (1.4–4.4)*	107/2951 (3.6%)		1.3 (1.1–1.8)*
District		< 0.001			< 0.001	
Eastern	28/4662 (0.6%)		Reference*	109/4662 (2.3%)		Reference*
Middle	10/1042 (1.0%)		2.1 (1.1–3.7)*	44/1042 (4.2%)		2.0 (1.5–2.6)*
Western	14/498 (2.8%)		3.2 (1.7–6.1)*	38/498 (7.6%)		3.2 (2.4–4.4)*
Resident place		0.78			0.82	
City	27/3376 (0.8%)		Reference	105/3376 (3.1%)		Reference
Rural	25/2826 (0.9%)		1.5 (0.9–2.6)	86/2826 (3.0%)		1.0 (0.8–1.3)
BCG vaccination history		0.98			0.07	
Yes	51/6086 (0.8%)		–	186/6086 (3.1%)		Reference
No	1/116 (0.9%)		–	5/116 (4.3%)		1.2 (0.7–2.1)
Presence of BCG scar		0.79			0.77	
Yes	49/5895 (0.8%)		Reference	183/5895 (3.1%)		Reference
No	3/307 (1.0%)		2.0 (0.8–5.0)	8/307 (2.6%)		0.9 (0.5–1.6)
Exposure level		< 0.001			< 0.001	
No reported contact risk	41/5833 (0.7%)		Reference*	151/5833 (2.6%)		Reference*
Non-household contact risk	6/274 (2.2%)		1.5 (0.6–3.6)*	23/274 (8.4%)		2.7 (1.9–3.9)*
Household contact risk	5/95 (5.3%)		6.1 (2.7–13.9)*	17/95 (17.9%)		6.9 (4.5–10.7)*

Data are n/N (%),unless otherwise indicated. n/N (%) is number positive /number with test results (proportion positive)

*BCG* Bacille Calmette Guerin, *IGRA* interferon-gamma release assay, *TST* tuberculin skin test, *OR* odd ratio, *CI* confidence interval

^#^: Univariable analysis; *: multivariable analysis, controlling for variables with p < 0.05 in univariable analysis. p values from the multivariable analysis for IGRA positivity was 0.02,0.005, 0.001 for age, district and exposure level respectively, for TST positivity was 0.13, < 0.001, < 0.001 for age, district and exposure level respectively

Agreement between results of the TST and IGRA was low, especially in children with no risk (κ = 0.15). Among children with no contact risk, 97.3% agreement between IGRA and TST were observed, including 97.0% positive results for both tests and 0.3% negative results for both tests. Similar results were observed in RID children with non-household or household contact risk and contrary results were observed in children with active TB (Table 3, Additional file 1: Tables S1 and S2). Compared with IGRA − /TST − results, a contact risk was significantly associated with IGRA+/TST+ results. The positive proportion and OR value for either IGRA− /TST+  or IGRA+ /TST− showed increasing trends with increased exposure risk. When IGRA and TST were discordant, TST was more likely to be positive and IGRA negative among children with RID, while TST negative and IGRA positive among children with active TB (Additional file 1: Table S1). Similar trends were found using 5 mm and six spots as the cut-off points for the TST and IGRA, respectively (Additional file 1: Table S2).

**Table 3:** Analysis of concordance and discordance between IGRA and TST

Risk group	IGRA+/TST+ n (%)	IGRA−/TST+ n(%)	IGRA+/TST− n(%)	IGRA−/TST− n(%)	*Kappa* value	*P* value	Ratio of	OR(95%CI)		
							TST+/IGRA− and TST−/IGRA+	IGRA+/TST+ vs. IGRA−/TST−	IGRA+/TST− vs. IGRA−/TST−	IGRA−/TST+ vs. IGRA−/TST−
No reported contact risk (n = 5833)	15 (0.3%)	136 (2.3%)	26 (0.4%)	5656 (97.0%)	0.15	< 0.001	5.2	Reference	Reference	Reference
Non-household contact risk (n = 274)	6 (2.2%)	17 (6.2%)	0 (0)	251 (91.6%)	0.39	< 0.001	–	9.0 (3.5–23.4)	1.0 (0.99–1.0)	2.8 (1.7–4.7)
Household contact risk (n = 95)	4 (4.2%)	13 (13.7%)	1 (1.1%)	77 (81.1%)	0.31	< 0.001	13	19.6 (6.4–60.4)	2.8 (0.4–21.1)	7.0 (3.8–12.9)

TST using 10 mm as a cutoff and IGRA using 8 spots as a cutoff

*IGRA* interferon-gamma release assay, *TST* tuberculin skin test, *RID* respiratory and infectious diseases, *OR* odds ratio, *CI* confidence interval

**Fig. 2:**
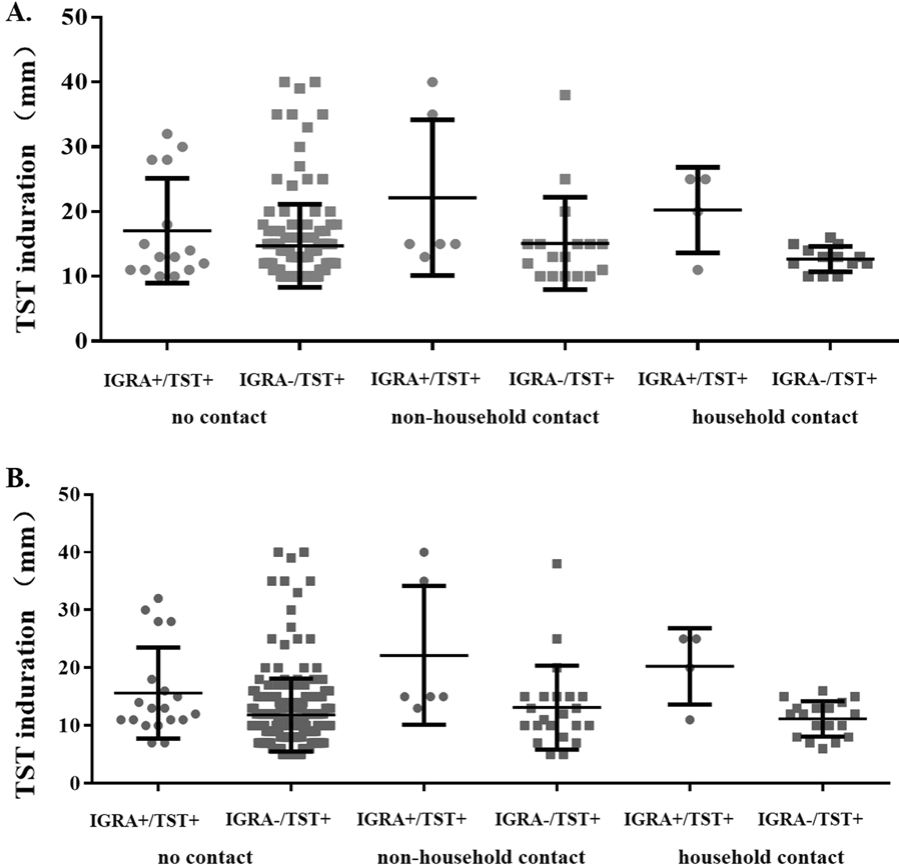
TST indurations between children with IGRA+/TST+ and IGRA−/TST+ results. **A** TST using 10 mm as a cutoff and IGRA using 8 spots as a cutoff. **B** TST using 5 mm as a cutoff and IGRA using 6 spots as a cutoff

**Fig. 3:**
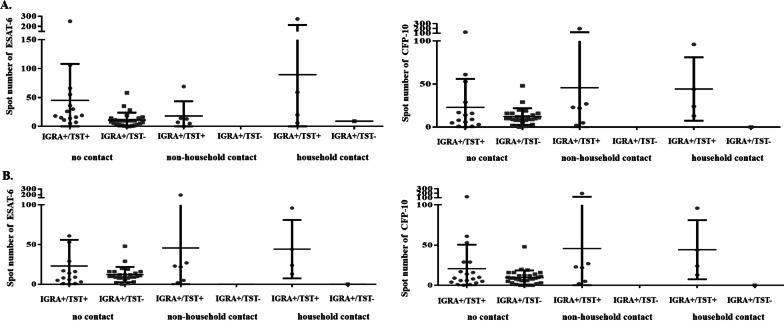
Spot numbers of IGRA between children with IGRA+/TST+ and IGRA+/TST− results. **A** TST using 10 mm as a cutoff and IGRA using 8 spots as a cutoff. **B** TST using 5 mm as a cutoff and IGRA using 6 spots as a cutoff

We calculated the parameters of immune responses measured by the IGRA and TST. When the induration diameter for the TST was compared between children with IGRA+/TST+ and those with IGRA− /TST+  results (using 10 mm as the cut-off value), children with IGRA+/TST+ results had larger indurations (*P* = 0.02) (Fig. 2A, Additional file 1: Table S3). Larger TST indurations were also found in children with IGRA+/TST+ than in those with IGRA− /TST+  results, using 5 mm as the cut-off value (Fig. 2B, Additional file 1: Table S4). Because of the limited number of children with positive IGRA results, the number of IFN-γ secreting T-cells responding to early-secreted antigenic target 6-kDa protein (ESAT-6) and culture filtrate protein 10 (CFP-10) could only be calculated in children with no reported contact risk. The findings showed that spot numbers were also much higher in children with IGRA+/TST+ than in children with IGRA+ /TST− results, using either 10 mm or 5 mm as the cut off value (Fig. 3 and Additional file 1: Table S5). On average, the IGRA+ /TST− results were slightly above the cutoff point: 10 (3–14) for ESAT-6, 10 (8–16) for CFP-10, respectively. The results for children with active TB are shown in Additional file 1: Tables S6–S8.

## Discussion

The risk of infection with MTB among children depends on several factors, one of the most important being close contact with a patient who has active TB. Therefore, development of an effective screening strategy in children with a contact history to active TB is critical to controlling the progression of disease. Children with an IGRA+/TST+ result usually have great clinical importance in confirming LTBI. Consistently, in our study, children with IGRA+/TST+ results were most significantly associated with a contact risk and had stronger responses than those with IGRA− /TST+  or IGRA+ /TST− results. These findings suggest that children with IGRA+/TST+ results can be diagnosed with LTBI and consequently, an evaluation for TB preventive treatment should be given in this group.

In the present study, we detected increased proportions of positive IGRA results and high OR values in children with household or non-household contact risk. Population-based multicenter studies performed in China have revealed that individuals with baseline results of IGRA+ /TST− showed a similar or higher incidence of active TB than those with baseline results IGRA+/TST+ or IGRA− /TST+  , either within the first 2 years of follow-up [12] or 5 years of follow-up [13]. Data from in our study which showed that discordance favored IGRA+/TST− in those with active TB whereas among those without active TB discordance favors TST+/IGRA−, suggested higher sensitivity and specificity of IGRA than that of TST. Considering the high specificity of IGRA reported in children [14] and higher sensitivity for identifying recent infection [15], an IGRA+ /TST− result is a critical reference for starting LTBI management.

We observed that the proportion of IGRA− /TST+  results increased with household exposure, which suggested that the TST response is related to exposure or infection to some extent. However, the increase of IGRA− /TST+  (from 2.3 to 13.7%) was much more significant than that of IGRA+/TST− (from 0.4% to 1.1%). Apart from the increased real infection rate, there may be some other factors associated with the IGRA−/TST+ result, including the BCG vaccination and NTM infection. As is shown in Additional file 1: Table S2, the mean TST induration in children with IGRA−/TST+ results were around 10 mm, a borderline result may suggest false positive response. A population-based, multicenter, cohort study demonstrated that TST results could be affected by multiple factors such as BCG vaccination, which might overestimate the prevalence of LTBI in China [16]. As BCG has been included in the national immunization program, all the newborn children should having got BCG vaccination theoretically in China. The influence of BCG vaccination on the specificity of TST results might explain the discordance observed in our study population. Non-tuberculous mycobacteria (NTM) infection is an emerging health problem among children [17]. An increase in NTM isolate prevalence has recently been detected in China [18]. Although the NTM prevalence in children has not been well measured in China, data from our hospital suggest that 13.3% of isolates from mycobacterial culture-positive pediatric patients were identified as NTM (data not published). Based on our findings, the IGRA might be used, in preference to the TST, as the initial diagnostic test for children with symptoms of RID or those with a contact risk.

Considering the cost of IGRA, the two-step approach (TST testing followed by IGRA if TST is positive to rule out false positive and improve specificity) might be performed for children during population-based LTBI screening, such as TB infection screening in school in China. One study analyzed the cost effectiveness of LTBI testing among Chinese adolescents and pointed out that the two-step approach yielded more cost savings than using IGRA alone (RMB 9181 vs. RMB 30,460 per positive) [19]. In addition, the high specificity of IGRA will allow for targeting of resources to the population most likely to benefit from LTBI therapy among those identified with LTBI using the TST only.

As the most frequently discordant, the IGRA− /TST+  results add complexity to LTBI diagnosis in children. According to our findings, additional clinical information should be used to confirm MTB infection status in children with IGRA− /TST+  results. A comprehensive diagnostic criterion should be set up using such items as contact risk, age, and epidemiological rate of TB in the residential area. IGRA− /TST+  results in children with a contact risk can be considered a reference for starting LTBI management. However, in our study, most IGRA− /TST+  results (136/166, 81.9%) were present in children with no risk or BCG vaccination. When balancing the benefit and risk, preventive treatment is not recommended in children with IGRA− /TST+  results and no exposure risk, especially those with borderline results.

A concern is that this two-step approach may miss children with IGRA+ /TST− results; however, the percentage of children with IGRA+ /TST− results is low. In healthy and BCG-vaccinated children, IGRA+ /TST− results are rare. We found that among 26 children with IGRA+ /TST− results and no contact risk, 18 had fewer than 15 spots, which was slightly higher than the cut-off values. This weak response might be explained as higher sensitivity or false positivity; this requires further study.

Another important finding of this study is that among children with no contact history, we identified 15 children with IGRA+/TST+ results and 26 children with IGRA+ /TST− results. Among them, most of the children aged 5 years or more and have entered school, participated in more community activities. Therefore, they are at higher risk of having positive results. This suggests that certain contact risks and a large number of infectious index patients remain unidentified, and the transmission routes are still ambiguous. School age children has been neglected and much of the tuberculosis burden in this age group occurs from school outbreaks. Nearly half of the new pediatric TB cases registered in the National Disease Surveillance Information Management System in China were students. TB could easily spread among students due to the highly crowded environment and the relatively weaker immune system. Although the positivity rate of IGRA and TST was low in this study, the screening strategy play an important role on the positive finding children with contact risk and those with active TB. So the screening strategy in students during their first year of admission to kindergarten or schools are necessary. This will be an important step in China's efforts to control childhood tuberculosis.

In addition, when using a two-step approach in contact tracing studies, the possibility that a TST can boost subsequent IGRA results should be considered. Pai et al. summarized the IGRA guidelines and recommendations and pointed out that a few country guidelines acknowledge this possibility [8]. Some specific recommendations have been made, including that the IGRA should be performed on the same day as the TST reading or within 3 days of TST placement.

Both the IGRA and TST have limited value in distinguishing LTBI from active TB and anticipating progression from latency to active disease. Considering the operational difficulties and the cost, IGRA is not an ideal screening tool in high-burden and resource-limited countries. Studies have therefore aimed to identify other MTB proteins, apart from ESAT-6 and CFP-10, that may serve as potential candidates for inclusion in immunodiagnostic tests for active TB or LTBI [20]. Several host response-based transcriptional signatures have been described for anticipating progression from latent to active disease [21, 22]; however, multicohort studies are needed to validate the value of these when translated to clinical practice.

Our study has several limitations that should be kept in mind when interpreting our results. First, because all enrolled participants were children admitted to our hospital, our study population was not entirely representative of the general population or hospitalized children in China. Children admitted to our hospital may have more severe or complicated conditions than children admitted to smaller regional hospitals. Whether these factors can affect the results of either test is unclear. A community study would provide stronger evidence for a given testing algorithm. The strength of this study lies in our findings being able to provide interim guidance, while awaiting stronger data from cohort studies. Second, it was difficult to determine whether any children had ever received a TST before being admitted to our hospital; thus, we could not ascertain whether the higher TST positivity rate was owing to a booster effect in some patients. This may partly explain the IGRA−/TST+ association with exposure observed in this study.

## Conclusion

Our results suggest that positive IGRA results in children with a contact risk can serve as a critical reference for starting LTBI management. The IGRA can be used, in preference to the TST, as the initial diagnostic test for children with an infection risk.

## Supplementary Information

**Additional file 1.** Additional tables.

## Data Availability

The datasets used and/or analyzed during the current study are available from the corresponding author on reasonable request.

## Abbreviations

LTBI: Latent tuberculosis infection
TST: Tuberculin skin test
IGRA: Interferon-gamma release assay
RID: Respiratory infectious diseases
ESAT-6: Early-secreted antigenic target 6-kDa protein
CFP-10: Culture filtrate protein 10
TB: Tuberculosis
MTB: *Mycobacterium tuberculosis*
BCG: Bacillus Calmette-Guérin
PPD: Purified protein derivative
