# Supplementary material for: Tuberculosis infection screening in children with close contact: a hospital-based study
Source: BMC Infect Dis. 2021 Aug 13;21:815. doi: 10.1186/s12879-021-06480-2 (PMC8364055; doi:10.1186/s12879-021-06480-2)
Supplement: Supplementary file 1 — Additional file 1. Additional tables. [file 12879_2021_6480_MOESM1_ESM.docx]

Table S1 Analysis of discordant results between IGRA and TST in children with active tuberculosis at different risk levels

| Risk group | IGRA+/TST+  n(%) | IGRA-/TST+  n(%) | IGRA+/TST-  n(%) | IGRA-/TST-  n(%) | *Kappa* value | *P* value | OR(95%CI) | | |
| --- | --- | --- | --- | --- | --- | --- | --- | --- | --- |
|  |  |  |  |  |  |  | IGRA+/TST+  vs. IGRA-/TST- | IGRA+/TST- vs. IGRA-/TST- | IGRA-/TST+ vs. IGRA-/TST- |
| non-household contact risk | 28(65.1%) | 2(4.7%) | 11(25.6%) | 2(4.7%) | 0.11 | 0.37 | reference | reference | reference |
| household contact risk | 21(50.0%) | 2(4.8%) | 17(40.5%) | 2(4.8%) | 0.02 | 0.84 | 0.8(0.1-5.8) | 1.5(0.2-12.6) | 1.0(0.1-16.0) |

IGRA: interferon-gamma release assay; TST: tuberculin skin test; OR: odd ratio; CI: confidence interval.

TST using 10 mm as a cutoff and IGRA using 8 spots as a cutoff

Table S2 Analysis of discordant results between IGRA and TST at different risk levels

| Risk group | IGRA+/TST+  n(%) | IGRA-/TST+  n(%) | IGRA+/TST-  n(%) | IGRA-/TST-  n(%) | *Kappa* value | *P* value | OR(95%CI) | | |
| --- | --- | --- | --- | --- | --- | --- | --- | --- | --- |
|  |  |  |  |  |  |  | IGRA+/TST+  vs. IGRA-/TST- | IGRA+/TST- vs. IGRA-/TST- | IGRA-/TST+ vs. IGRA-/TST- |
| Children with RID |  |  |  |  |  |  |  |  |  |
| no reported contact risk | 19(0.3%) | 217(3.7%) | 32(0.5%) | 5565(95.4%) | 0.12 | <0.001 | reference | reference | reference |
| non-household contact risk | 6(2.2%) | 22(8.0%) | 0(0) | 246(89.8%) | 0.33 | <0.001 | 7.1(2.8-18.0) | 0.99(0.99-1.0) | 2.3(1.5-3.6) |
| household contact risk | 4(4.2%) | 18(18.9%) | 1(1.1%) | 72(75.8%) | 0.23 | 0.002 | 16.3(5.4-49.0) | 2.4(0.3-17.9) | 6.4(3.8-10.9) |
| Children with TB |  |  |  |  |  |  |  |  |  |
| non-household contact risk | 35(81.4%) | 2(4.7%) | 5(11.6%) | 1(2.3%) | 0.14 | 0.32 | reference | reference | reference |
| household contact risk | 35(83.3%) | 3(7.1%) | 3(7.1%) | 1(2.4%) | 0.17 | 0.27 | 1.0(0.1-16.0) | 0.6(0.1-13.6) | 1.5(0.1-40.6) |
| Total | 99(1.6%) | 262(4.2%) | 41(0.7%) | 5885(93.6%) | 0.38 | <0.001 | - | - | - |

RID: respiratory and infectious diseases; TB: tuberculosis

TST using 5 mm as a cutoff and IGRA using 6 spots as a cutoff

Table S3 Comparison of TST induration between children with IGRA+/TST+ and IGRA-/TST+ results

| Risk group | TST induration for  IGRA+/TST+ (mm) | TST induration for  IGRA-/TST+ (mm) | *P* value |
| --- | --- | --- | --- |
| Children with RID | 15.0(11.5-26.5) | 13.0(10.5-15.5) | 0.02 |
| no reported contact risk | 13.0(11.0-28.0) | 13.0(10.0-15.0) | 0.40 |
| non-household contact risk | 15.0(14.5-36.3) | 15.0(10.5-22.5) | 0.14 |
| household contact risk | 22.5(13.3-25.0) | 13.0(12.0-15.0) | 0.10 |

RID: respiratory and infectious diseases; IGRA: interferon-gamma release assay; TST: tuberculin skin test.

The data was shown as median value and interquartile ranges.

TST using 10mm as a cutoff and IGRA using 8 spots as a cutoff

Table S4 Comparison of TST induration between children with IGRA+/TST+ and IGRA-/TST+ results

| Risk group | IGRA+/TST+ (mm) | IGRA-/TST+ (mm) | *P* value |
| --- | --- | --- | --- |
| Children with RID | 15.0 (11.0-25.0) | 10.0 (8.0-14.0) | <0.001 |
| no reported contact risk | 13.0 (11.0-18.0) | 10.0 (8.0-14.0) | 0.02 |
| non-household contact risk | 15.0 (14.5-36.3) | 12.0 (10.0-15.0) | 0.03 |
| household contact risk | 22.5 (13.3-25.0) | 8.0 (12.0-13.8) | <0.001 |
| Children with TB |  |  |  |
| non-household contact risk | 14.0 (12.0-16.0) | 15.0 | 0.30 |
| household contact risk | 11.0 (8.0-15.0) | 10.0 | 0.22 |

RID: respiratory and infectious diseases

TST using 5 mm as a cutoff and IGRA using 6 spots as a cutoff

Table S5 Comparison of spot numbers of IGRA between children with IGRA+/TST+ and IGRA+/TST- results

| Risk group | ESAT-6 | | | CFP-10 | | |
| --- | --- | --- | --- | --- | --- | --- |
|  | IGRA+/TST+ (n) | IGRA+/TST- (n) | *P* value | IGRA+/TST+ (n) | IGRA+/TST- (n) | *P* value |
| Children with RID | 18.0(11.0-59.0) | 9.0 (3.0-14.0) | 0.015 | 16.0 (5.0-44.0) | 10.0 (8.0-16.0) | 0.03 |
| no reported contact risk | 19.0 (11.0-55.0) | 10.0 (3.0-14.0) | 0.011 | 9.0 (5.0-29.0) | 10.0 (8.0-16.0) | 0.13 |
| non-household contact risk | 10.0 (7.0-14.0) | - | - | 14.0 (5.0-27.0) | - | - |
| household contact risk | 40.0 (20.0-59.0) | 9.0 | - | 34.0 (24.0-44.0) | 0 | - |

RID: respiratory and infectious diseases; IGRA: interferon-gamma release assay; TST: tuberculin skin test; ESAT: early-secreted antigenic target 6-kDa protein; CFP-10: culture filtrate protein 10.

“-” means uncalculated data because no children or only one child in this group.

TST using 10mm as a cutoff and IGRA using 8 spots as a cutoff

Table S6 Comparison of spot numbers of IGRA between children with IGRA+/TST+ and IGRA+/TST- results

| Risk group | ESAT-6 | | | CFP-10 | | |
| --- | --- | --- | --- | --- | --- | --- |
|  | IGRA+/TST+ (n) | IGRA+/TST- (n) | *P* value | IGRA+/TST+ (n) | IGRA+/TST- (n) | *P* value |
| Children with RID |  |  |  |  |  |  |
| no reported contact risk | 18.0(11.0-36.0) | 6.0(2.3-10.0) | 0.009 | 9.0(4.0-29.0) | 8.5(3.5-12.0) | 0.05 |
| non-household contact risk | 10.0(3.8-27.8) | - | - | 22.5(4.3-69.3) | - | - |
| household contact risk | 39.5(9.5-219.5) | 9.0 | - | 34.0(15.8-83.0) | 0 | - |
| Children with TB |  |  |  |  |  |  |
| non-household contact risk | 34.0(9.0-68.0) | 33.0(17.0-154.0) | 0.94 | 19.0(4.0-104.0) | 16.0(13.0-253.0) | 0.58 |
| household contact risk | 43.0(20.0-102.0) | 33.0(10.0-113.5) | 0.79 | 49.0(17.0-85.0) | 20.0(10.5-72.0) | 0.57 |

RID: respiratory and infectious diseases;

“-” means uncalculated data because no children with IGRA+/TST- results in community-contacts with RID or the Mann-Whitney U test cannot performed because only one children had IGRA+/TST- results in household-contacts with RID.

TST using 5 mm as a cutoff and IGRA using 6 spots as a cutoff

Table S7 Comparison of TST induration between children with IGRA+/TST+ and IGRA-/TST+ results in children with active tuberculosis

| Risk group | TST induration for  IGRA+/TST+ (mm) | TST induration for  IGRA-/TST+ (mm) | *P* value |
| --- | --- | --- | --- |
| non-household contact risk | 15.0(12.0-17.0) | 15.0 | 0.82 |
| household contact risk | 15.0(12.0-20.0) | 10.5 | 0.09 |

IGRA: interferon-gamma release assay; TST: tuberculin skin test.

The data was shown as median value and interquartile ranges.

TST using 10 mm as a cutoff and IGRA using 8 spots as a cutoff

Table S8 Comparison of spot numbers of IGRA between children with IGRA+/TST+ and IGRA+/TST- results in children with active tuberculosis

| Risk group | ESAT-6 | | | CFP-10 | | |
| --- | --- | --- | --- | --- | --- | --- |
|  | IGRA+/TST+ (n) | IGRA+/TST- (n) | *P* value | IGRA+/TST+ (n) | IGRA+/TST- (n) | *P* value |
| non-household contact risk | 35.0(12.3-67.0) | 33.0(11.0-76.0) | 0.92 | 15.0(4.3-102.4) | 52.0(16.0-163.0) | 0.48 |
| household contact risk | 57.0(24.5-125.0) | 33.0(12.5-113.5) | 0.45 | 52.0(18.5-100.5) | 26.0(12.5-72.0) | 0.21 |

TB: tuberculosis; IGRA: interferon-gamma release assay; TST: tuberculin skin test; ESAT: early-secreted antigenic target 6-kDa protein; CFP-10: culture filtrate protein 10.

“-” means uncalculated data because no children with IGRA+/TST- results in community-contacts with RID or the Mann-Whitney U test cannot performed because only one children had IGRA+/TST- results in household-contacts with RID.

TST using 10mm as a cutoff and IGRA using 8 spots as a cutoff
